# Is the Verification Phase a Suitable Criterion for the Determination of Maximum Oxygen Uptake in Patients with Heart Failure and Reduced Ejection Fraction? A Validation Study

**DOI:** 10.3390/ijerph20042764

**Published:** 2023-02-04

**Authors:** Agustín Manresa-Rocamora, Laura Fuertes-Kenneally, Carles Blasco-Peris, Noemí Sempere-Ruiz, José Manuel Sarabia, Vicente Climent-Paya

**Affiliations:** 1 Institute for Health and Biomedical Research of Alicante (ISABIAL), 03010 Alicante, Spain; 2 Department of Sport Sciences, Sports Research Centre, Miguel Hernández University of Elche, 03202 Elche, Spain; 3 Cardiology Department, Dr. Balmis General University Hospital, 03010 Alicante, Spain; 4 Department of Physical Education and Sport, University of Valencia, 46010 Valencia, Spain

**Keywords:** cardiorespiratory fitness, VO_2_ max, HFrEF, exercise testing, respiratory exchange ratio, gradual exercise test, VO_2_ peak

## Abstract

The verification phase (VP) has been proposed as an alternative to the traditional criteria used for the determination of the maximum oxygen uptake (VO_2_ max) in several populations. Nonetheless, its validity in patients with heart failure with reduced ejection fraction (HFrEF) remains unclear. Therefore, the aim of this study was to analyse whether the VP is a safe and suitable method to determine the VO_2_ max in patients with HFrEF. Adult male and female patients with HFrEF performed a ramp-incremental phase (IP), followed by a submaximal constant VP (i.e., 95% of the maximal workload during the IP) on a cycle ergometer. A 5-min active recovery period (i.e., 10 W) was performed between the two exercise phases. Group (i.e., median values) and individual comparisons were performed. VO_2_ max was confirmed when there was a difference of ≤ 3% in peak oxygen uptake (VO_2_ peak) values between the two exercise phases. Twenty-one patients (13 males) were finally included. There were no adverse events during the VP. Group comparisons showed no differences in the absolute and relative VO_2_ peak values between both exercise phases (*p* = 0.557 and *p* = 0.400, respectively). The results did not change when only male or female patients were included. In contrast, individual comparisons showed that the VO_2_ max was confirmed in 11 patients (52.4%) and not confirmed in 10 (47.6%). The submaximal VP is a safe and suitable method for the determination of the VO_2_ max in patients with HFrEF. In addition, an individual approach should be used because group comparisons could mask individual differences.

## 1. Introduction

Heart failure with reduced ejection fraction (HFrEF) is a cardiovascular disorder characterised by symptoms of breathlessness, fluid retention, and exercise intolerance [1,2,3]. The maximal ramp or step incremental exercise test, coupled with breath-by-breath and gas exchange measurements, is widely used in patients with HFrEF to measure maximum oxygen uptake (VO_2_ max) and for risk stratification [4,5,6]. VO_2_ max is defined as the physiological limit of oxygen utilisation [7] and is considered a strong predictor of mortality in patients with HFrEF [8,9]. In fact, VO_2_ max is considered a prognostic factor in advanced heart failure and is currently used as a key criterion for the selection of candidates for heart transplantation (i.e., ≤14 mL·kg^−1^·min^−1^) [4].

The measurement of VO_2_ max requires the patient to perform a maximal exercise effort (i.e., volitional exhaustion) and thus might be substantially underestimated due to muscle fatigue, breathlessness, and reduced motivation (i.e., submaximal exercise test). In these circumstances, the peak oxygen uptake (VO_2_ peak) instead of the VO_2_ max is obtained. Consequently, it is important to determine the criteria to accurately categorise an effort as maximal. The primary criterion used to verify maximal exercise relies on the presence of the oxygen uptake (VO_2_) plateau, which is defined as no increase in VO_2_ despite an increase in workload rate [10,11]. Nonetheless, data indicate that only a small percentage of VO_2_ assessments actually exhibit a VO_2_ plateau [12,13,14,15], supporting the argument that this physiological phenomenon is not necessary to acutely determine the VO_2_ max. Thus, secondary criteria such as the value of the respiratory exchange ratio (RER)—which is the most frequently used variable in cardiac patients—age-predicted maximal heart rate (HR), or blood lactate concentrations are commonly used to verify that a maximal exercise effort has been achieved [13,16,17]. However, evidence suggests that these criteria lack validity since they can be met with either a maximal or submaximal exercise effort or even, not reached at all despite a maximal effort [11,18,19]. In summary, traditional criteria (i.e., including both primary and secondary) are not reliable methods to ensure that the VO_2_ max is reached at the end of an incremental exercise test [20].

In an attempt to overcome the shortcomings of traditional criteria, a new criterion for the determination of the VO_2_ max has emerged, known as the verification phase (VP) [13]. The VP is a constant-load phase performed following the incremental phase (IP) and a short recovery period (e.g., 3–5 min). Other protocols have also been used previously [20]. Regarding its intensity, it can be performed either above (i.e., supramaximal verification phase) or below (i.e., submaximal verification phase) the peak work rate attained in the previous IP [10,15]. There is evidence demonstrating that the VP is an adequate standard for validating the VO_2_ max in healthy individuals [21] and patients with a wide range of pathologies [22].

In this regard, Bowen et al. [23] investigated whether the submaximal VP was a valid method to determine the VO_2_ max in patients with HFrEF. According to the authors, the VP was well tolerated by patients with HFrEF, and its precision was greater than that of secondary criteria (i.e., RER). Nonetheless, only male patients were included, and further research is needed to determine whether VP is suitable and well tolerated by female patients with HFrEF. Furthermore, although both group and individual comparisons can be used to validate the VO_2_ max, group comparisons could mask individual differences between the VO_2_ peak values attained in each exercise phase [19,20]. Also, the clinical utility of the exercise test is its application to the individual rather than the group. For these reasons, individual comparisons are more useful than group comparisons [24]. In order to perform these individual comparisons and assess whether or not the VO_2_ max was reached, a standard cut-off point should be established, preferentially using relative differences (e.g., ≤3%) [20]. In contrast, Bowen et al. [23], who included group and individual comparisons, carried out statistical comparisons. In this study, the VO_2_ max was confirmed when statistical significance was not reached (*p* > 0.050). Nonetheless, the use of statistical comparisons is a flawed approach because it is designed to detect differences and depends on the sample size [25]. Thus, the use of different approaches to conduct individual comparisons and confirm the VO_2_ max warrants future studies in patients with HFrEF.

Therefore, the main purpose of the current study was to investigate the utility of the submaximal VP for validating the VO_2_ max in male and female patients with HFrEF. In addition, we compared the level of agreement between the VP and traditional (i.e., RER) criteria for the determination of the VO_2_ max. Based on previous evidence, we hypothesised that the submaximal VP would be an adequate criterion to verify the VO_2_ max in male and female patients with HFrEF when an individual approach is used, and no agreement would be found between both criteria.

## 2. Materials and Methods

### 2.1. Patients

Participants needed to fulfil the following inclusion criteria to be eligible: (a) male or female aged between 50 and 70 years old; (b) diagnosed with HFrEF (left ventricular ejection fraction < 50%); (c) stable phase of the disease with no recent hospitalisation or visit to the emergency department due to heart failure (within the last six months before the beginning of the study); (d) New York Heart Association (NYHA) functional class I, II, or III; (e) under treatment with B-blockers; and (f) sedentary (i.e., not involved in exercise training for six months). The exclusion criteria were: (a) use of intravenous diuretics in the last six months; (b) unstable angina or evidence of severe ventricular arrhythmia; (c) atrial fibrillation; (d) supraventricular arrhythmias; (e) chronic obstructive pulmonary disease; (f) recent of haemoglobin concentrations outside optimal parameters (13–16.5 g·dL^−1^); (g) physical limitations that impeded the completion of the ergometry; and (h) the presence of ischaemia, arrhythmias, or high frequency of ectopic heartbeats. All patients were fully informed and signed the informed consent before any procedure related to the study was performed. The protocol of this study was approved by the competent ethics committee of the host institution (PI2021-177).

### 2.2. Measurements

Participants performed a symptom-limited exercise test which comprised two phases; (a) the ramp-incremental exercise phase (i.e., IP); and (b) the steady-state exercise phase (i.e., VP). The test was carried out on an electromagnetically braked cycle ergometer (SanaBike 500 easy, Truchtelfinger, Germany). Before the start of the IP, a 3 min warm-up at 10 W and a cadence of 50 revolutions per minute (rpm) was performed. The IP ended when the patient reached volitional exhaustion or was unable to maintain a cadence of at least 45 rpm. The exercise test was terminated, and the VP was not carried out in the presence of symptoms of ischaemia or multifocal ectopic heartbeats (symptom-limited). Otherwise, a free-cadence recovery period of 5 min at 10 W was performed after finishing the IP. Subsequently, the VP was carried out at 95% of the maximum power reached during the IP. Throughout the protocol, gas exchange was recorded with the Metalyzer 3B gas analyser (CORTEX Biophysik, Leipzig, Germany), and HR was monitored with a 12-lead electrocardiograph. Patients were asked to fast (at least three hours prior to the test), as well as to refrain from strenuous physical activity (24 h), alcohol, and smoking (three hours prior).

### 2.3. Data and Statistical Analyses

Gas exchange and ventilatory variables were analysed to remove atypical breaths (four standard deviations from the local mean) due to swallows, coughs, and so on [26]. VO_2_ peak was defined as the highest VO_2_ occurring during each exercise phase (i.e., IP and VP). VO_2_ peak, as well as the remaining ventilatory variables obtained at exercise peak (i.e., VCO_2_, oxygen pulse, RER, VE, VE/VCO_2_, and VE/VO_2_), were identified using a 12-breath rolling average [23]. Breathing frequency and HR were averaged over 10 s.

Data are displayed as median (25th and 75th percentiles) and frequency (percentage) for continuous and categorical variables, respectively, unless stated otherwise. Overall, the Fisher-Pitman permutation test [27] and the non-parametric 95% confidence interval (CI) of the difference [28] were used to conduct between-phase comparisons (i.e., VP vs. IP). The Bland-Altman plot was used to test the agreement between VO_2_ peak values measured during the IP and VP.

Individual comparisons between VO_2_ peak values reached during the two exercise phases were also conducted. In this regard, the IP-derived VO_2_ peak was confirmed (i.e., VO_2_ max) if the difference with the VP-derived VO_2_ peak value was ≤ 3%. Afterwards, patients were classified into two groups, depending on whether IP-derived VO_2_ max values were confirmed or not. Fisher’s exact test, Mann-Whitney test, and Bonett-Price 95% CI were used to conduct between-group comparisons (i.e., confirmed vs. not confirmed groups).

The traditional criterion (i.e., RER peak ≥ 1.10) was also used to verify VO_2_ max [17]. Compared to the VP, the results were classified as follows: (a) agreement, RER ≥ 1.10 and VO_2_ max confirmed by VP or RER < 1.10 and VO_2_ max not confirmed by VP; (b) false positive, RER ≥ 1.10 and VO_2_ max not confirmed by VP; and (c) false negative, RER < 1.10 and VO_2_ max confirmed by VP. The Kappa index was used to analyse the degree of agreement between the two criteria (i.e., RER vs. VP). All tests were two-sided, and *p* values ≤ 0.050 were considered significant. All analyses were performed using STATA software (version 16.0; Stata Corp LLC, College Station, TX, USA).

## 3. Results

### 3.1. Patients

Thirty patients with HFrEF (22 males; 73.3%) fulfilled the inclusion criteria to be eligible to participate in the current study. Nonetheless, we excluded a total of eight patients (26.6%) because the VP was considered contraindicated (i.e., symptoms of ischaemia, arrhythmias, or high frequency of ectopic heartbeats during the IP). Moreover, one patient (3.3%) did not complete the VP due to knee pain and was also excluded from the analysis. All excluded patients were male. Therefore, 21 patients (13 males; 61.9%) were finally included. The characteristics of these patients are shown in Table 1. No adverse events occurred during the exercise tests. The median age was 64.0 years (57.5; 68.5), and the median left ventricular ejection fraction was 39.1% (33.0; 43.1). Ischemic etiology was the cause of HFrEF in almost half of the patients. Most of the included patients were smokers (81%).

### 3.2. Group Comparisons

**Figure 1:**
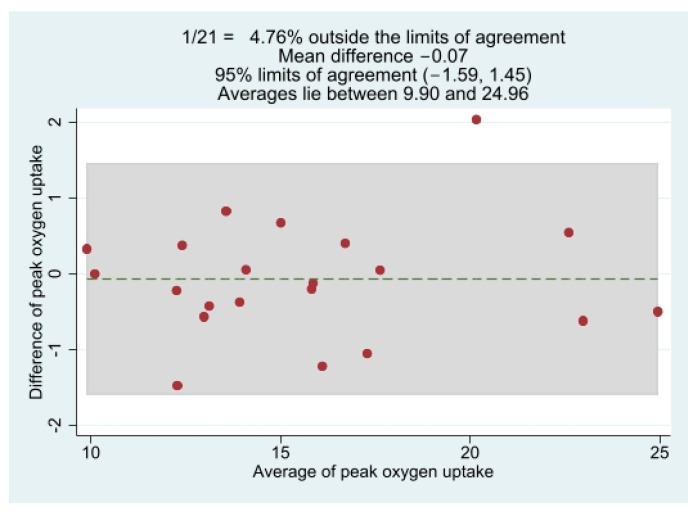
Bland-Altman plot for relative peak oxygen uptake response between the two exercise phases. Dashed line represents the mean bias, and highlighted zone indices are the limits of agreement (mean ± 1.96 standard deviation).

Descriptive group data from the IP and VP, as well as between-phase comparisons, are presented in Table 2. The median peak work rate during the IP was 55.0 W (46.5; 92.5). The absolute and relative VO_2_ peak values did not differ between exercise phases (*p* = 0.557 and *p* = 0.400, respectively). RER and VE/VCO_2_ peak values were lower and higher, respectively, in the VP than in the IP (*p* = 0.004 and *p* = 0.003). The results did not change when exclusively male or female patients were included in the analyses. Figure 1 shows the Bland-Altman plot for the relative VO_2_ peak. The mean difference between both exercise phases was −0.07 mL·kg^−1^·min^−1^, while the lower and upper limits of agreement were −1.59 mL·kg^−1^·min^−1^ and 1.45 mL·kg^−1^·min^−1^, respectively.

### 3.3. Individual Comparisons

An IP-derived VO_2_ peak was confirmed (i.e., VO_2_ max) in 11 (52.4%) patients and not confirmed (i.e., VO_2_ peak) in 10 (47.6%). Regarding the patients in whom the VO_2_ peak was attained, five showed higher IP-derived VO_2_ peak values and five showed lower IP-derived VO_2_ peak values, compared with the VP-derived VO_2_ peak value. As to the patient characteristics, comparisons showed that the proportion of smokers was higher (*p* = 0.035) in the confirmed group (100%) than in the not confirmed group (60%). Interestingly, the percentage of female participants did not differ between groups (*p* = 0.999). Moreover, there were no between-group differences in any of the remaining analysed variables (*p* > 0.050) (see Table 1).

### 3.4. Confirmed and Not Confirmed Groups

The patients’ responses to both exercise phases in the confirmed and not confirmed groups can be found in Table S1. Between-phase comparisons showed the same results as those found when all patients had been included (see Table 2). On the other hand, between-group comparisons during each exercise phase are shown in Table S2. Although no statistically significant differences were found (*p* > 0.050), the relative VO_2_ peak value was higher in the confirmed group compared to the not confirmed group both in the IP (2.62 mL·kg^−1^·min^−1^ [95%CI = −2.38 to 7.62]; *p* = 0.305) and the VP (2.35 mL·kg^−1^·min^−1^ [95%CI = −2.90 to 7.60]; *p* = 0.380).

### 3.5. Agreement between the Traditional and Verification Phase Criteria

When the traditional criterion (i.e., RER peak) was used, VO_2_ max was confirmed in 10 patients and VO_2_ peak was attained in 11 patients. The median VO_2_ peak values in the confirmed and not-confirmed groups during the IP were 16.2 mL·kg^−1^·min^−1^ (13.0; 19.9) and 14.1 mL·kg^−1^·min^−1^ (13.0; 17.6), while the median values during the VP were 15.7 mL·kg^−1^·min^−1^ (13.6; 21.6) and 13.7 mL·kg^−1^·min^−1^ (11.5; 16.8), respectively. Regarding the agreement between the two criteria for the determination of VO_2_ max, there were 10 agreements (47.6%), six false negative cases (28.6%), and five false positive cases (23.8%). Moreover, the Kappa index showed that there was no significant agreement between both criteria (Kappa = −0.045; *p* = 0.583).

## 4. Discussion

The main objective of this study was to investigate whether the submaximal VP is a safe and reliable method to validate VO_2_ max in male and female patients with HFrEF. To accomplish this, we used both group and individual approaches. Additionally, we investigated the agreement between the RER and VP criteria to determine VO_2_ max.

Regarding our results, no adverse events were observed during the exercise tests, suggesting that VP is a safe method for determining VO_2_ max in patients with HFrEF. In agreement with our findings, Bowen et al. [23] also reported no adverse events in patients with HFrEF. There is also previous evidence showing that the use of the VP was well-tolerated in patients with other diseases, such as cancer [29], prehypertension [30], and metabolic syndrome [31], who are normally sedentary and not familiarised with high-intensity exercise. Nonetheless, it should be noted that, in the current study, patients who had a high risk of adverse events (those who presented symptoms of ischaemia or ectopic heartbeats during the IP) were exempt from performing the VP. Moreover, several patients had difficulty cycling since they were unfamiliar with the cycle ergometer. In this regard, Manresa-Rocamora et al. [32] reported a greater improvement in the VO_2_ max after an exercise-based cardiac rehabilitation programme in studies that conducted the incremental exercise test on a cycle ergometer compared to studies that used a treadmill in patients with coronary artery disease. The lack of habituation to the cycle ergometer could explain, in part, the higher training-induced effect found in these studies, seeing as their baseline VO_2_ max results were worse than those who used a treadmill. Therefore, a familiarisation period should be performed before conducting the incremental exercise test to avoid terminating the test due to peripheral fatigue.

As for the use of individual versus group comparisons for the analysis of VP, contradictory findings were obtained based on the type of approach used to conduct the analyses. Group comparisons showed that both exercise phases (i.e., IP and VP) led to similar median VO_2_ peak values. These results did not change when only male or female patients were included in the analysis. Therefore, based on this approach, the VO_2_ peak values reached during the IP can be considered as maximal (i.e., VO_2_ max) in all patients. This finding is in line with those of Murias et al. [33] and Bowen et al. [23] in healthy males and patients with HFrEF, respectively. In this regard, Murias et al. [33], who did not conduct individual comparisons, concluded that both the submaximal VP (i.e., 85% of peak power output) and supramaximal VP (i.e., 105% of peak power output) were not necessary to confirm the VO_2_ max values reached during the preceding IP. In the same line, Astorino and Emma [22] and Costa et al. [34], who respectively conducted a review and a meta-analysis (54 studies), reported no differences in mean VO_2_ peak values between the two exercise phases in a sizable number of studies conducted with healthy adults and individuals with pathology. Previous studies also failed to find differences between both exercise phases in endurance-trained athletes [35,36]. Interestingly, in line with our results, Costa et al. [34] found that the sex of the participants did not influence their results and reported no differences in the aggregate VO_2_ peak values in male and female participants. In contrast to these findings, Moreno-Cabañas et al. [31] and Schaun et al. [37] found higher mean VO_2_ peaks during the VP than during the IP in male and female older adults with obesity and hypertension, respectively. It should be noted that a supramaximal VP (i.e., constant load and multistage) preceded by a passive recovery period (i.e., 10–15 min) in the seated position was conducted, which could explain in part these controversial findings. In contrast, Costa et al. [34] reported in their meta-analysis no differences in mean VO_2_ peaks regardless of the VP intensity (i.e., submaximal vs. supramaximal), type of recovery (i.e., active vs. passive), verification timing (i.e., same day vs. different day), and verification phase duration (e.g., less than 80 s) in apparently healthy adults. Bhammar and Chien [30], who conducted a supramaximal VP, also found no differences in VO_2_ peak values in adults with prehypertension. Therefore, our findings and previous evidence support that the submaximal VP is not necessary to confirm VO_2_ max when group comparisons are used, while the utility of the supramaximal VP, which could lead to controversial findings, in patients with HFrEF requires future study. Nonetheless, the achievement of a VO_2_ peak is an individual phenomenon and group comparisons may cloud individual differences.

In relation to individual comparisons, our results showed that VO_2_ max was confirmed by the VP in 52% of the patients with HFrEF, while a VO_2_ peak was attained (i.e., the individual between-phase difference in VO_2_ peak values higher than 3%) in the remaining patients (48%). Moreover, the percentage of female patients was the same in the confirmed and not confirmed groups, suggesting that individual comparisons could be used in both male and female patients. Nonetheless, the low number of female patients included warrants future studies to confirm our results. Similarly, Bowen et al. [23], who only recruited male patients, found that the VO_2_ max was confirmed in 58% of the patients with HFrEF included in their study. It should be highlighted that, in contrast to our study, statistical comparisons between both exercise phases were performed to conduct individual comparisons and validate the VO_2_ max. In conclusion, regardless of the criteria used to carry out individual comparisons, an individual approach should be prioritised to determine the VO_2_ max in patients with HFrEF, in accordance with previous reports in the literature [24,37].

On the other hand, we found no difference in median VO_2_ peak values between the two exercise phases in the confirmed and not confirmed groups, which also agrees with the results of Bowen et al. [23]. In the same line, the current and the former study showed no difference between the two groups in aggregate VO_2_ peak values reached during the IP. However, although statistical significance was not reached, both studies showed that the group VO_2_ peak values achieved during the IP were higher in the confirmed group (15.9 and 15.1 mL·kg^−^^1^·min^−^^1^) than in the not confirmed group (13.3 and 13.7 mL·kg^−^^1^·min^−^^1^). These findings seem to support a greater difference in VO_2_ peak values between both exercise phases (i.e., VO_2_ peak attained) in patients with lower cardiorespiratory fitness. Furthermore, Moreno-Cabañas et al. [31], who included older and less physically fit participants with obesity, observed higher VP-derived VO_2_ peak in 40% of the participants, while Wood et al. [38], who recruited younger and fitter patients with obesity, only found a difference in VO_2_ peak values between the two exercise phases in 15% of the participants. Therefore, our findings and previous evidence seem to support that patients with lower cardiorespiratory fitness may show a greater difference in VO_2_ peaks between the two exercise phases, with the use of the VP being even more important for the validation of the VO_2_ max in this group of patients.

Finally, regarding the comparison between traditional criteria (i.e., RER) and the VP for VO_2_ max determination, we found no agreement between both methods, which is similar to previous evidence [23]. Interestingly, based on the RER criterion, the VO_2_ max was confirmed in five patients who showed higher VO_2_ peaks during the VP compared with the IP (i.e., false positive). There is evidence showing that the RER criterion can be reached at submaximal intensities (e.g., 80% VO_2_ max) [11,18], which concurs with our findings. Moreover, Bowen et al. [23] showed a direct relationship between RER and workload increase in patients with HFrEF. In the same line, Moreno-Cabañas et al. [31] observed that the VO_2_ plateau was not reliable for determining the VO_2_ max in participants with obesity. Therefore, the results of the current and previous studies confirm that traditional criteria (e.g., RER and VO_2_ plateau) should not be due to their lack of validity to verify the VO_2_ max.

## 5. Limitations

Some limitations should be mentioned. First, we did not perform a familiarisation period with the equipment (e.g., cycle ergometer) and, consequently, some patients showed difficulty cycling. Future studies conducted with patients who are sedentary should include a familiarisation phase before starting the study protocol. Second, there was an uneven sex distribution among the participants (i.e., 13 males and 8 females). Therefore, to support our findings, additional research including more female patients with HFrEF should be conducted. Third, no prior power analysis was conducted to estimate the optimum number of patients the study should include.

## 6. Conclusions

The submaximal VP is a safe and suitable method to determine the VO_2_ max in patients with HFrEF. When comparing both exercise phases, an individual approach is preferable, seeing as aggregate comparisons could mask patients who showed differences in VO_2_ peaks between both exercise phases.

## Figures and Tables

**Table 1:** Baseline participant characteristics.

Variable	n = 21	Confirmed Group (n = 11)	Not Confirmed Group (n = 10)	*p*
Age, years	64.0 (57.5; 68.5)	64.0 (56.0; 66.0)	65.0 (58.0; 69.3)	0.717
Height, cm	164 (158; 171)	164 (157; 168)	164 (159; 174)	0.617
Weight, kg	70.0 (66.5; 87.3)	72.0 (64.0; 88.5)	69.6 (67.8; 80.6)	0.850
Body mass index, kg/m^2^	27.8 (24.8; 31.6)	27.8 (25.1; 32.0)	27.7 (24.1; 31.4)	0.557
LVEF, %	39.1 (33.0; 43.1)	39.0 (33.0; 45.0)	37.5 (32.3; 43.0)	0.414
Male (%)	13 (61.9)	7 (63.6)	6 (60.0)	0.999
Ischemic etiology (%)	10 (47.7)	6 (54.6)	4 (40.0)	0.670
Diabetes mellitus (%)	9 (42.9)	4 (36.4)	5 (50.0)	0.670
Hypertension (%)	9 (42.9)	4 (36.4)	5 (50.0)	0.670
Dyslipidaemia (%)	11 (52.4)	6 (54.6)	5 (50.0)	0.999
Smokers (%)	17 (81.0)	11 (100)	6 (60.0)	**0.035**
ICD (%)	9 (42.9)	5 (45.5)	4 (40.0)	0.999
Drug therapy:				
ACEI/ARBs (%)	8 (38.1)	4 (36.4)	4 (40.0)	0.999
ARNI (Sac/Val) (%)	13 (61.9)	7 (63.4)	6 (60.0)	0.999
MRA (%)	17 (81.0)	9 (81.2)	8 (80.0)	0.999
Antiplatelet (%)	8 (38.1)	4 (36.4)	4 (40.0)	0.999
Anticoagulants (%)	2 (9.5)	2 (18.2)	0 (0)	0.476
Diuretics (%)	3 (14.3)	1 (9.1)	2 (20.0)	0.586

ACEI, Angiotensin-converting enzyme inhibitors; ARNI (Sac/Val), angiotensin receptor-neprilysin inhibitor (sacubitril/valsartan); ICD, implantable cardioverter defibrillator; LVEF, left ventricular ejection fraction; MRA, mineralocorticoid receptor antagonist. Data are presented as median (25th and 75th percentiles) or frequency (percentage); *p* values refer to between-group differences; bold values refer to statistical significance (*p* ≤ 0.050).

**Table 2:** Cardiopulmonary responses to the two exercise phases and between-phase comparisons (n = 21).

Variable	IP	VP	Difference (95% CI)	*p*
Duration, min	8.5 (7.3; 12.5)	2.8 (2.1; 3.5)	−7.25 (−9.30 to −5.20)	**<0.001**
HR peak, beats·min^−1^	112.0 (108.0; 127.0)	109.0 (105.5; 128.0)	−1.00 (−5.92 to 4.92)	0.720
RER peak	1.10 (1.04; 1.12)	1.00 (0.95; 1.08)	−0.08 (−0.13 to −0.02)	**0.004**
VO_2_ peak, ml·min^−1^	1.02 (0.89; 1.59)	1.01 (0.89; 1.58)	−0.007 (−0.026 to 0.012)	0.557
VO_2_ peak, ml·kg^−1^·min^−1^	14.7 (13.1; 17.7)	15.3 (12.7; 17.3)	−0.09 (−0.35 to 0.18)	0.400
O_2_ pulse, ml·beat^−1^	10.0 (8.0; 13.0)	10.0 (8.0; 13.0)	0.00 (−0.002 to 0.002)	0.999
VE peak, l·min^−1^	44.9 (36.4; 64.0)	43.6 (35.8; 62.4)	0.40 (−3.64 to 4.44)	0.550
VE/VO_2_ peak	36.5 (32.9; 40.9)	35.6 (32.5; 39.1)	0.90 (−2.73 to 4.53)	0.338
VE/VCO_2_ peak	35.5 (32.4; 36.7)	36.4 (33.4; 39.9)	2.90 (1.33 to 4.47)	**0.003**
BF peak, breaths·min^−1^	36.0 (29.5; 41.0)	37.0 (30.5; 41.5)	0.50 (−2.51 to 3.51)	0.746

BF peak, peak breath frequency; CI, confidence interval; HR peak, peak heart rate; IP, incremental phase; O_2_ pulse, Oxygen pulse; RER peak, peak respiratory exchange ratio, VE peak, peak ventilation; VE/VCO_2_ peak, peak ventilatory equivalent for carbon dioxide; VE/VO_2_ peak, peak ventilatory equivalent for oxygen; VO_2_ peak, peak oxygen uptake; VP, verification phase. Exercise phase data are presented as median (25th and 75th percentiles); *p* values refer to within-subject comparisons (VP vs. IP); bold values refer to statistical significance (*p* ≤ 0.050).

## Data Availability

The datasets generated from the current study are available from the corresponding author upon reasonable request.

## Supplementary Materials

The following supporting information can be downloaded at: https://www.mdpi.com/article/10.3390/ijerph20042764/s1, Table S1: Cardiopulmonary responses to the two exercise phases in the confirmed and not confirmed groups, and between-phase comparisons; Table S2: Between-group comparisons during the two exercise phases.
