# Supplementary material for: Is the Verification Phase a Suitable Criterion for the Determination of Maximum Oxygen Uptake in Patients with Heart Failure and Reduced Ejection Fraction? A Validation Study
Source: Int J Environ Res Public Health. 2023 Feb 4;20(4):2764. doi: 10.3390/ijerph20042764 (PMC9956911; doi:10.3390/ijerph20042764)
Supplement: Supplementary file 1 [file ijerph-20-02764-s001.zip › ijerph-2188350-supplementary.pdf]

International Journal of
Environmental Research and Public Health

Table S1. Cardiopulmonary responses to the two exercise phases in the confirmed and not confirmed groups, and between-phase comparisons.

	Confirmed group (n = 11)				Not confirmed group (n = 10)			
**Variable**	**IP**	**VP**	**Difference (95% CI)**	***p***	**IP**	**VP**	**Difference (95% CI)**	***p***
Peak work rate, W	63.0 (47.0; 100.0)				48.0 (45.0; 88.5)			
Duration, min	9.0 (8.0; 13.0)	2.8 (2.0; 3.5)	–7.25 (–9.30 to –5.20)	**<0.001**	8.3 (6.3; 12.4)	3.0 (2.2; 3.5)	–5.78 (–8.86 to –2.69)	**0.002**
HR peak, beats·min^{-1}	115.0 (110.0; 137.0)	117.0 (106.0; 137.0)	–1.00 (–6.92 to 4.92)	0.720	110.0 (106.0; 120.5)	107.0 (103.5; 118.8)	–2.50 (–4.79 to 0.21)	0.328
RER peak	1.07 (1.05; 1.12)	0.99 (0.95; 1.04)	–0.08 (–0.13 to –0.02)	**0.004**	1.10 (0.98; 1.20)	1.00 (0.92; 1.12)	–0.07 (–0.10 to –0.03)	**0.006**
VO_2 peak, ml·min^{-1}	1.15 (0.94; 1.64)	1.13 (0.93; 1.65)	–0.006 (–0.03 to 0.01)	0.557	0.97 (0.86; 1.36)	0.97 (0.82; 1.39)	–0.01 (–0.08 to 0.07)	0.703
VO_2 peak, ml·kg^{-1}·min^{-1}	15.9 (14.1; 22.3)	15.8 (13.7; 22.7)	–0.09 (–0.35 to 0.18)	0.400	13.3 (12.8; 17.0)	13.5 (12.3; 15.8)	–0.12 (–1.08 to 0.84)	0.881
O_2 pulse, ml·beat^{-1}	11.0 (8.0; 13.0)	11.0 (8.0; 13.0)	0.00 (–0.002 to 0.002)	0.999	9.5 (7.8; 12.3)	8.5 (7.8; 12.3)	–0.50 (–1.39 to 0.39)	0.500
VE peak, l·min^{-1}	46.2 (40.4; 67.9)	47.0 (39.7; 63.5)	0.40 (–3.64 to 4.44)	0.550	38.7 (36.2; 62.1)	38.2 (32.8; 58.4)	–1.40 (–5.76 to 2.96)	0.744
VE/VO_2 peak	37.0 (32.2; 40.3)	36.5 (34.3; 42.5)	0.90 (–2.73 to 4.53)	0.338	36.3 (33.2; 41.6)	34.5 (32.1; 38.4)	–0.95 (–1.91 to 0.01)	0.613
VE/VCO_2 peak	35.9 (33.1; 36.6)	38.3 (35.9; 40.6)	2.90 (1.33 to 4.47)	**0.003**	35.1 (31.6; 37.2)	35.2 (32.8; 39.9)	0.90 (–0.37 to 2.17)	0.125
BF peak, breaths·min^{-1}	38.0 (29.0; 43.0)	41.0 (31.0; 44.0)	0.50 (–2.51 to 3.51)	0.746	35.0 (31.0; 39.3)	36.0 (29.8; 39.0)	0.00 (–2.48 to 2.48)	0.950

BF peak, peak breath frequency; CI, confidence interval; HR peak, peak heart rate; IP, incremental exercise phase; O_2 pulse, Oxygen pulse; RER peak, peak respiratory exchange ratio, VE peak, peak ventilation; VE/VCO_2 peak, peak ventilatory equivalent for carbon dioxide; VE/VO_2 peak, peak ventilatory equivalent for oxygen; VO_2 peak, peak oxygen uptake; VP, verification phase.
Exercise phase data are presented as median (25th and 75th percentiles); *p* values refer to within-subject comparisons (VP vs. IP); bold values refer to statistical significance ($p \leq 0.050$).

International Journal of
Environmental Research and Public Health

Table S2. Between-group comparisons during the two exercise phases.

	IP		VP	
**Variable**	**Difference (95% CI)**	***p***	**Difference (95% CI)**	***p***
Peak work rate, W	15.0 (–19.8 to 49.8)	0.374		
Duration, min	0.75 (–3.64 to 5.14)	0.738	–0.25 (–1.23 to 0.73)	0.618
HR peak, beats·min^{-1}	5.0 (–12.2 to 22.2)	0.568	10.0 (–8.9 to 29.0)	0.301
RER peak	–0.03 (–0.18 to 0.12)	0.686	–0.02 (–0.13 to 0.10)	0.795
VO_2 peak, ml·min^{-1}	0.18 (–0.29 to 0.65)	0.459	0.16 (–0.32 to 0.64)	0.517
VO_2 peak, ml·kg^{-1}·min^{-1}	2.62 (–2.38 to 7.62)	0.305	2.35 (–2.90 to 7.60)	0.380
O_2 pulse, ml·beat^{-1}	1.50 (–2.19 to 5.19)	0.426	2.50 (–1.19 to 6.19)	0.184
VE peak, l·min^{-1}	7.50 (–12.47 to 27.47)	0.462	8.85 (–10.39 to 28.09)	0.367
VE/VO_2 peak	0.75 (–5.16 to 6.66)	0.804	2.00 (–3.75 to 7.75)	0.496
VE/VCO_2 peak	0.85 (–2.85 to 4.55)	0.652	3.10 (–0.99 to 7.19)	0.138
BF peak, breaths·min^{-1}	3.00 (–6.03 to 12.03)	0.515	5.00 (–3.12 to 13.12)	0.130

BF peak, peak breath frequency; CI, confidence interval; HR peak, peak heart rate; IP, incremental exercise phase; O_2 pulse, Oxygen pulse; RER peak, peak respiratory exchange ratio, VE peak, peak ventilation; VE/VCO_2 peak, peak ventilatory equivalent for carbon dioxide; VE/VO_2 peak, peak ventilatory equivalent for oxygen; VO_2 peak, peak oxygen uptake; VP, verification phase.
p values refer to between-subject comparisons (confirmed vs. not confirmed groups).
